# Physical bacteria–neuron proximity and early cellular responses: a conceptual perspective

**DOI:** 10.3389/fnins.2026.1844325

**Published:** 2026-06-23

**Authors:** Rosa Rivero Guillén

**Affiliations:** Independent Researcher, Barcelona, Spain

**Keywords:** bacteria–neuron proximity, cellular responses, extracellular vesicles, intracellular CA^2+^ dynamics, mechanotransduction, membrane perturbation, membrane-associated signaling, microbiota–gut–brain axis

## Abstract

Recent experimental observations obtained in reduced *in vitro* systems have reported direct proximity between bacteria and neuronal cells associated with intracellular Ca^2+^ dynamics and transcriptomic alterations. In particular, studies involving *Lactiplantibacillus plantarum* and primary cortical neuronal cultures have described bacterial adhesion to neuronal surfaces together with modulation of neuroplasticity-associated proteins and gene networks related to cellular signaling and neuronal regulation. Current models of the microbiota–gut–brain axis primarily emphasize indirect communication mediated through metabolites, immune pathways, neuroendocrine signaling, vagal pathways, extracellular vesicles, and soluble mediators. Although these mechanisms possess substantial explanatory value, certain early cellular responses observed under conditions of direct bacteria–neuron proximity may not be fully interpretable exclusively through soluble signaling mechanisms. This manuscript proposes a conceptual perspective in which the neuronal membrane is considered a dynamic cellular interface potentially sensitive to localized mechanical, physicochemical, or membrane-associated perturbations generated under conditions of direct biological proximity. Within this context, intracellular Ca^2+^ dynamics are interpreted as possible early cellular responses that may emerge in association with membrane-associated perturbation. Potential candidate mechanisms including mechanosensitive ion channels, localized membrane perturbation, adhesion-associated signaling, cytoskeletal remodeling, membrane reorganization, and local physicochemical microenvironmental alterations are discussed together with alternative explanations involving soluble mediators, immune activation, extracellular vesicles, osmotic or ionic perturbations, and generalized cellular stress responses. Importantly, the currently available evidence derives exclusively from reduced experimental systems and does not establish physiological relevance or demonstrated neuromodulation *in vivo*. Rather than proposing an alternative model of microbiota–brain communication, this perspective aims to refine interpretation of emerging neurobacterial interface observations by defining experimentally testable hypotheses and mechanistically plausible questions for future investigation.

## Introduction

Communication between the microbiota and the nervous system has emerged as a central topic in contemporary physiology, neuroscience, and host–microbe interaction research. Current models of the microbiota–gut–brain axis primarily emphasize indirect communication pathways mediated through microbial metabolites, immune signaling, neuroendocrine modulation, vagal pathways, extracellular vesicles, and soluble signaling molecules (Dinan and Cryan, 2017; Cryan et al., 2019; Kaelberer et al., 2018; Needham et al., 2020). These mechanisms possess substantial explanatory value across both physiological and pathological contexts and have significantly expanded current understanding of microbiota-associated neural regulation.

At the same time, observations obtained in multiple biological systems suggest that local cellular responses may be influenced by both chemical and physicochemical conditions present at the cellular interface (Levin, 2021; Sun et al., 2024). Within this context, reduced experimental systems may provide an opportunity to explore whether direct biological proximity between microorganisms and neuronal cells could contribute to localized early cellular responses under controlled experimental conditions.

From a cellular physiology perspective, biological membranes are dynamic structures capable of responding to local physicochemical and mechanical perturbations through multiple membrane-associated processes (Ingber, 2006; Ranade et al., 2015). In parallel, evidence suggests that electrical or physicochemical microenvironmental conditions may influence microbial localization and certain host–microbe interactions under specific biological contexts (Prindle et al., 2015; Sun et al., 2024).

Within this framework, Lombardo-Hernandez et al. (2025) described a reduced *in vitro* neurobacterial interface in which *Lactiplantibacillus plantarum* physically adhered to primary rat cortical neurons without penetrating the neuronal soma. Under these controlled experimental conditions, bacterial proximity was associated with intracellular Ca^2+^ dynamics, modulation of neuroplasticity-associated proteins including Synapsin I and phosphorylated CREB (pCREB), and transcriptomic alterations involving gene categories associated with neuronal-related functions and cellular signaling (Lombardo-Hernandez et al., 2025).

Importantly, these observations derive from controlled experimental systems and do not establish direct bacteria–neuron communication under physiological *in vivo* conditions. Nevertheless, the reproducibility of the observed responses, their concentration dependence, the differential effects observed between viable and inactivated bacteria, and the presence of concurrent intracellular alterations suggest that certain cellular responses associated with direct bacteria–neuron proximity may not be fully explained by generalized stochastic perturbation or classical soluble signaling mechanisms alone.

The aim of this manuscript is not to propose an alternative model to the established microbiota–gut–brain axis, but rather to explore whether membrane-associated biological proximity may represent an additional experimental variable worth considering in interpretation of specific observations involving bacteria–neuron proximity in reduced *in vitro* systems.

## Current experimental observations

Recent experimental studies conducted in reduced *in vitro* systems have described direct proximity between bacteria and neuronal cells under controlled experimental conditions (Lombardo-Hernandez et al., 2025).

Among the most relevant examples, Lombardo-Hernandez et al. (2025) developed an experimental neurobacterial interface using *Lactiplantibacillus plantarum* and primary rat cortical neuronal cultures. Using confocal microscopy, the authors observed bacterial adhesion to neuronal surfaces and neuritic processes without evidence of bacterial penetration into the neuronal soma.

Functional analysis using real-time calcium imaging demonstrated increases in intracellular Ca^2+^ dynamics following bacterial exposure. Importantly, the magnitude of these responses appeared dependent on bacterial concentration and active bacterial metabolism, since inactivated bacteria induced substantially weaker intracellular responses (Lombardo-Hernandez et al., 2025). These observations may reflect not only metabolic inactivity, but also differences in bacterial surface organization, membrane-associated adhesins, physicochemical interface properties, or local microenvironmental perturbations capable of influencing cellular responses at the membrane interface.

In addition to intracellular Ca^2+^ dynamics, the study reported alterations in proteins associated with neuronal plasticity, including Synapsin I and phosphorylated CREB (pCREB). Transcriptomic analysis further revealed changes in gene expression involving gene categories associated with neuronal-related functions, cellular signaling, and membrane-associated processes (Lombardo-Hernandez et al., 2025).

Importantly, several characteristics of the reported observations—including reproducibility, concentration dependence, differential responses between viable and inactivated bacteria, modulation of neuroplasticity-associated proteins, and transcriptomic alterations—argue against a purely stochastic or entirely non-specific perturbation model, even though the underlying mechanistic basis remains unresolved.

Together, these findings support the possibility that direct bacteria–neuron proximity may be associated with measurable early cellular responses in reduced experimental systems.

However, several important limitations substantially constrain interpretation.

First, intracellular Ca^2+^ dynamics may emerge in association with multiple overlapping cellular processes, including mechanotransduction, membrane perturbation, cellular stress responses, immune receptor activation, metabolic adaptation, and intracellular signaling pathways (Berridge et al., 2003; Clapham, 2007). Accordingly, the currently available observations do not establish a specific functional signaling program or demonstrated neuromodulatory mechanism.

Second, transcriptomic alterations alone do not establish functional neuronal modulation. Although changes in neuroplasticity-associated proteins were observed, direct electrophysiological measurements evaluating membrane potential, neuronal excitability, synaptic integration, or network-level functional responses were not performed.

Third, the experimental conditions do not reproduce the physiological complexity of *in vivo* systems, including epithelial barriers, mucus-layer compartmentalization, immune surveillance, extracellular matrix organization, enteric nervous system regulation, microbiota spatial segregation, and tissue-level architecture (Turner, 2009).

Accordingly, the current evidence should be interpreted within the context of controlled experimental systems that permit direct biological proximity between bacteria and neuronal cells.

## Cautious biophysical interpretation

The currently available observations raise the possibility that direct biological proximity between bacterial surfaces and neuronal membranes may contribute to early cellular responses under certain controlled experimental conditions.

One possible interpretation is that the neuronal membrane functions as a dynamic cellular interface responsive to localized physicochemical perturbations associated with biological proximity. Such perturbations could involve membrane deformation, alterations in lipid microdomains, receptor clustering, mechanosensitive activation, ionic or osmotic microenvironmental shifts, or cytoskeletal remodeling (Ingber, 2006).

Several candidate mechanisms could plausibly contribute to these early cellular responses. Mechanosensitive ion channels, including Piezo family members and transient receptor potential (TRP) channels such as TRPV and TRPA subtypes, are known to respond to subtle membrane perturbations, osmotic changes, membrane tension alterations, and mechanical stimuli (Ranade et al., 2015; Szczot et al., 2018; Venkatachalam and Montell, 2007). These pathways may therefore represent plausible candidates through which localized membrane perturbations could influence intracellular signaling under conditions of direct bacteria–membrane proximity.

Similarly, integrin-associated signaling and focal adhesion complexes may represent additional candidate pathways through which localized membrane interactions could influence intracellular signaling cascades and cytoskeletal dynamics. Alterations in lipid microdomains and receptor clustering may also contribute to localized signaling responses at the cellular interface.

In addition, bacterial surface-associated structures, membrane-associated adhesins, extracellular polymeric components, extracellular vesicles, or local physicochemical microenvironmental changes could contribute to membrane perturbations capable of influencing intracellular signaling without requiring direct cellular invasion.

Within this framework, intracellular Ca^2+^ dynamics may represent early integrative cellular responses associated with membrane perturbation, mechanotransduction, cellular adaptation, stress-associated responses, receptor-mediated activation, metabolic responses, or other overlapping intracellular processes (Berridge et al., 2003; Clapham, 2007). Although intrinsically non-specific, calcium signaling frequently functions as an early hub of cellular state regulation and adaptive response.

Accordingly, the observed intracellular Ca^2+^ alterations should not be interpreted as evidence of demonstrated neuronal communication, physiological neuromodulation, or validated functional integration within neuronal networks. Rather, they may indicate that direct biological proximity at the membrane interface can be associated with measurable early cellular responses under controlled experimental conditions.

**FIGURE 1:**
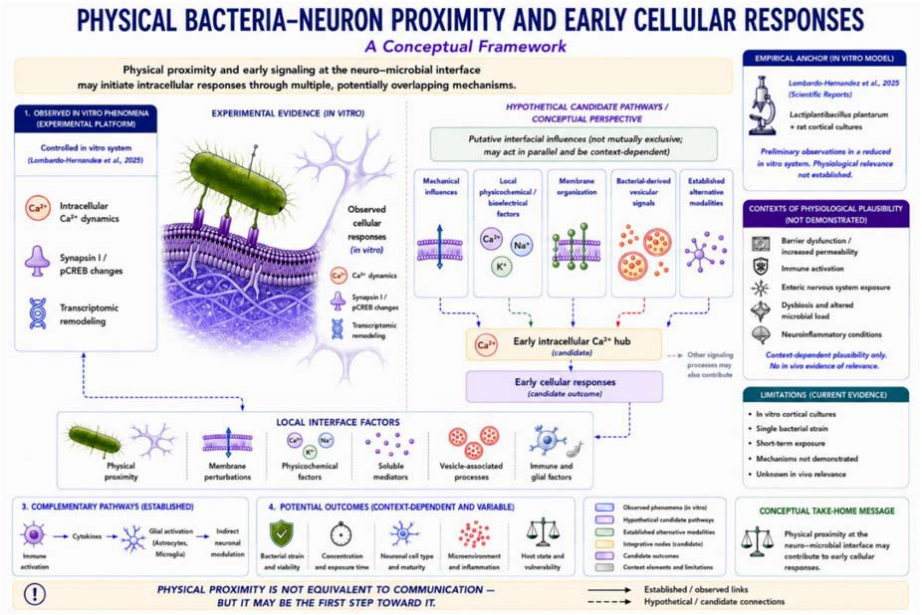
Conceptual framework illustrating experimentally observed phenomena and hypothetical candidate mechanisms associated with bacteria–neuron proximity in reduced in vitro systems.

A conceptual overview of the candidate mechanisms discussed throughout this framework is presented in Figure 1. The schematic summarizes hypothetical membrane-associated pathways, established complementary signaling modalities, and potential early intracellular responses associated with bacteria–neuron proximity in reduced experimental systems.

The left side summarizes experimentally observed findings reported by Lombardo-Hernandez et al. (2025), including bacterial adhesion to neuronal surfaces, intracellular Ca^2+^ dynamics, modulation of neuroplasticity-associated proteins, and transcriptomic remodeling.

The central and right sections outline hypothetical candidate pathways that may contribute to early cellular responses under conditions of direct bacteria–neuron proximity, including membrane-associated perturbations, local physicochemical and bioelectrical microenvironmental changes, mechanosensitive signaling, extracellular vesicle-associated processes, and established complementary signaling modalities.

Solid arrows indicate experimentally observed findings or biologically established associations. Dashed arrows indicate hypothetical or unresolved mechanistic relationships.

## Alternative hypotheses

The observations currently available may plausibly be interpreted through multiple non-exclusive mechanisms, all of which should be considered when evaluating bacteria–neuron proximity-associated cellular responses.

### Soluble molecule–mediated signaling

One possibility is that the observed intracellular responses are primarily mediated through soluble bacterial products rather than direct biological proximity itself. Microbial metabolites, secreted peptides, extracellular polymeric components, endotoxin-associated molecules, or extracellular vesicles may induce neuronal intracellular responses without requiring sustained membrane contact (Cryan et al., 2019).

Extracellular vesicles have emerged as important mediators of microbiota–host communication capable of transporting bioactive proteins, lipids, metabolites, and nucleic acids across biological interfaces while modulating neuroimmune and neurocellular responses (Morales Medina et al., 2022). Accordingly, some intracellular alterations observed in bacteria–neuron co-culture systems could theoretically result from localized soluble or vesicle-associated signaling processes occurring near the cellular interface.

### Pattern recognition receptor activation

Bacterial surface-associated molecules may also activate pattern recognition receptors, including Toll-like receptors (TLRs), NOD-like receptors, and related innate immune signaling pathways expressed by neuronal or glial cells. Activation of these pathways may induce intracellular cascades involving Ca^2+^ mobilization, inflammatory signaling, metabolic responses, and transcriptomic alterations independently of mechanobiological processes.

### Non-specific cellular stress responses

Another possible interpretation is that bacterial adhesion or localized biological proximity generates perturbations capable of inducing generalized cellular responses. These may include membrane stress, osmotic alterations, metabolic imbalance, pH shifts, membrane tension fluctuations, or local disturbances of membrane homeostasis that secondarily influence intracellular Ca^2+^ dynamics and transcriptional responses (Berridge et al., 2003; Clapham, 2007).

Importantly, generalized stress responses and intracellular signaling processes may partially overlap under conditions of localized biological perturbation.

### Contact-associated mechanotransduction

An additional hypothesis is that localized biological proximity contributes to early intracellular responses through mechanotransduction-associated pathways involving mechanosensitive ion channels, receptor clustering, focal adhesion-associated signaling, cytoskeletal remodeling, or local physicochemical microenvironmental perturbations (Ingber, 2006; Ranade et al., 2015; Sun et al., 2024).

Within this framework, physical proximity may contribute to early intracellular signaling dynamics without implying demonstrated neuronal communication or resolved physiological integration.

Importantly, these candidate mechanisms should not be interpreted as mutually exclusive explanatory models. Multiple processes may contribute simultaneously to the observed responses.

At the current level of evidence, however, the relative contribution of individual mechanisms remains unresolved and represents one of the principal interpretative limitations of the phenomenon under investigation.

## Biological interface

Current models of host–microbiota communication are predominantly based on chemical and biological signaling mechanisms, including microbial metabolites, neuroactive compounds, cytokines, extracellular vesicles, immune mediators, and neuroendocrine pathways (Rhee et al., 2009; Cryan et al., 2019). These mechanisms possess substantial explanatory value and remain central to current understanding of microbiota–host physiological regulation across multiple biological contexts.

At the same time, observations obtained in reduced experimental systems suggest that direct biological proximity between bacteria and neuronal cells may also be associated with measurable early intracellular responses (Lombardo-Hernandez et al., 2025). The functional interpretation of these observations remains unresolved and may depend on the contribution of different candidate mechanisms operating locally at the cellular interface.

From this perspective, the neuronal membrane may respond to local mechanical, ionic, metabolic, or physicochemical perturbations generated at the cell surface. Such perturbations may involve receptor clustering, mechanosensitive signaling, local ion fluxes, adhesion-associated dynamics, cytoskeletal remodeling, localized physicochemical microenvironmental alterations, or other membrane-associated processes (Ingber, 2006; Ranade et al., 2015).

Importantly, this potential sensitivity should be understood as a general property of membrane organization and cellular physiology rather than as a specialized biological system specifically evolved for microorganism detection.

Within this framework, direct biological proximity is not proposed as an alternative to established models of microbiota–gut–brain communication. Rather, it may represent an additional experimental variable worth considering when interpreting certain observations obtained in reduced bacteria–neuron co-culture systems.

This perspective is also compatible with the possibility that different cellular processes may contribute to local intracellular responses under specific experimental conditions. However, the current evidence remains insufficient to determine the relative contribution of these mechanisms.

## Limitations

This conceptual framework presents substantial experimental, mechanistic, and interpretative limitations that should be explicitly acknowledged.

First, the currently available evidence derives exclusively from reduced *in vitro* systems conducted under controlled experimental conditions that do not reproduce the physiological complexity of living tissues. These systems lack essential biological components including epithelial barriers, mucus-layer compartmentalization, immune surveillance, extracellular matrix organization, vascular regulation, enteric nervous system integration, and structured microbiota spatial organization (Turner, 2009; Cryan et al., 2019).

In addition, the bacteria-to-cell ratios employed in experimental co-culture systems may not accurately reflect physiological biological conditions and could potentially amplify intracellular responses associated with sustained artificial proximity between bacteria and neuronal cells.

A further limitation concerns interpretation of intracellular Ca^2+^ dynamics. Calcium responses may emerge in association with multiple overlapping cellular processes, including mechanotransduction, membrane perturbation, inflammatory activation, immune signaling, metabolic responses, and cellular stress (Berridge et al., 2003; Clapham, 2007). Accordingly, intracellular Ca^2+^ elevation alone cannot establish neuronal communication or physiological neuromodulation.

Importantly, direct electrophysiological measurements evaluating membrane potential, neuronal excitability, synaptic integration, network-level activity, or functional cellular responses have not been performed in the currently available experimental studies. Consequently, it remains impossible to determine whether the observed intracellular responses possess meaningful functional consequences at the neuronal systems level.

Another important limitation concerns the unresolved mechanistic basis underlying the observed responses. At the current level of evidence, it remains impossible to experimentally discriminate between the relative contribution of mechanotransduction-associated responses, soluble mediators, extracellular vesicle–associated signaling, immune receptor activation, physicochemical microenvironmental perturbations, or generalized cellular stress responses.

Furthermore, the physiological relevance of sustained direct bacteria–neuron proximity has not been established *in vivo*. Although specialized anatomical locations may permit close spatial relationships between microbial products and neuronal structures, the complex architecture of physiological tissues introduces multiple biological barriers, including epithelial layers, mucus barriers, extracellular matrices, immune surveillance systems, and spatial constraints that are not represented in simplified co-culture systems.

Certain pathological or altered biological contexts could theoretically increase the plausibility of localized bacteria–neuron proximity-associated phenomena. Such contexts may include epithelial barrier dysfunction, inflammatory states, dysbiosis-associated permeability alterations, enteric nervous system exposure, tissue injury, altered extracellular matrix organization, or neurodegenerative processes associated with disrupted tissue architecture.

Accordingly, the present framework should be interpreted as an experimentally constrained conceptual perspective intended to refine interpretation of emerging neurobacterial interface observations and organize experimentally testable hypotheses rather than establish demonstrated physiological mechanisms of microbiota–brain communication.

## Testability

The present conceptual framework is experimentally testable and should ultimately be evaluated through approaches capable of discriminating between the multiple candidate mechanisms potentially contributing to bacteria–neuron proximity-associated cellular responses.

A first experimental priority would involve evaluating the dependence of the observed intracellular responses on sustained proximity between bacteria and neuronal cells. If membrane-related mechanisms contribute significantly to the observed phenomena, experimental disruption of bacteria–neuron adhesion would be expected to reduce, alter, or spatially reorganize intracellular Ca^2+^ dynamics and downstream transcriptional responses. Experimental approaches involving adhesion-blocking strategies, modified bacterial surface structures, altered bacterial adhesins, or controlled physical separation systems may help clarify this question.

A second major priority concerns discrimination between direct contact-associated responses and soluble signaling-associated mechanisms. Experimental strategies involving conditioned media, bacterial supernatants, extracellular vesicle isolation, transwell systems, or localized microfluidic separation approaches could help determine whether the observed neuronal responses can be reproduced in the absence of sustained bacteria–neuron proximity (Cryan et al., 2019; Morales Medina et al., 2022).

Additional experiments involving controlled mechanical perturbation of neuronal membranes may further help evaluate the potential contribution of mechanotransduction-associated pathways. In this context, selective modulation of candidate mechanosensitive channels including Piezo family members or TRP-associated pathways could provide mechanistic insight into whether localized membrane perturbation contributes to intracellular signaling (Ranade et al., 2015; Szczot et al., 2018).

Complementary approaches evaluating lipid microdomain organization, receptor clustering, cytoskeletal remodeling, focal adhesion-associated signaling, local ion fluxes, or physicochemical microenvironmental alterations may further help clarify whether membrane perturbations participate in initiation or modulation of early cellular responses.

Direct electrophysiological evaluation represents another essential experimental requirement. Measurements involving membrane potential, neuronal excitability, synaptic integration, intracellular Ca^2+^ dynamics, firing behavior, and network-level activity would be necessary to determine whether the currently observed intracellular alterations possess meaningful functional consequences beyond early cellular responses.

Further investigation using more physiologically complex experimental systems would also be required. Reproducibility in neuronal organoids, gut–neuron co-culture systems, enteric nervous system models, neuroimmune interfaces, epithelial barrier systems, or *in vivo* experimental conditions would be necessary to evaluate the biological plausibility and potential physiological relevance of these phenomena within intact tissue environments.

Importantly, these experimental approaches would not only help determine whether direct bacteria–neuron proximity contributes to the observed phenomena, but also allow discrimination between alternative explanatory mechanisms involving soluble signaling, extracellular vesicle–associated communication, immune receptor activation, mechanotransduction-associated pathways, physicochemical microenvironmental perturbations, and generalized cellular stress responses.

Accordingly, the primary value of the present conceptual framework lies in its ability to generate experimentally distinguishable and mechanistically testable hypotheses capable of refining interpretation of emerging neurobacterial interface observations while maintaining clear physiological and interpretative boundaries.

## Conclusion

Recent experimental observations obtained in reduced *in vitro* systems suggest that direct proximity between bacteria and neuronal cells may be associated with measurable early intracellular responses including intracellular Ca^2+^ dynamics, modulation of neuroplasticity-associated proteins, and transcriptomic alterations (Lombardo-Hernandez et al., 2025).

Importantly, these observations do not demonstrate physiological neuromodulation, establish a resolved communication mechanism between bacteria and neurons, or confirm the existence of direct neurobacterial signaling under *in vivo* physiological conditions.

Nevertheless, the reproducibility of the reported findings, their dependence on bacterial viability and concentration, and the modulation of intracellular responses suggest that direct bacteria–neuron proximity may represent an additional experimental variable worth considering in the interpretation of certain bacteria–neuron co-culture phenomena.

Within this framework, the present manuscript proposes an experimentally testable and mechanistically open perspective in which localized membrane perturbations may be associated with early intracellular responses observed under controlled experimental conditions together with established soluble, immune-associated, vesicle-mediated, and neuroendocrine signaling mechanisms.

Potential candidate mechanisms involving mechanotransduction-associated pathways, adhesion-associated signaling, local physicochemical microenvironmental perturbations, receptor clustering, or membrane-related signaling processes remain speculative but biologically plausible and experimentally approachable.

Importantly, this perspective is not intended to replace current models of microbiota–gut–brain communication, which remain strongly supported by extensive evidence involving microbial metabolites, immune regulation, neuroendocrine pathways, extracellular vesicles, and vagal signaling (Cryan et al., 2019).

Rather, the principal value of the present framework lies in its ability to organize experimentally distinguishable and mechanistically testable hypotheses capable of refining interpretation of emerging neurobacterial interface observations while maintaining a clear distinction between experimentally observed phenomena, conceptual interpretation, and speculative mechanistic extrapolation.

Intracellular Ca^2+^ dynamics participate in multiple aspects of cellular physiology, including signaling responses associated with metabolism, mitochondrial activity, and adaptive cellular responses (Berridge et al., 2003; Clapham, 2007). Accordingly, the currently available observations may reflect localized cellular responses occurring at the neurobacterial interface without necessarily implying integrated microbiota–brain communication at the organismal level. Further investigation across progressively more complex biological systems will therefore be required before broader physiological interpretations can be considered.

## Data Availability

The original contributions presented in this study are included in this article/supplementary material, further inquiries can be directed to the corresponding author.
